# Dietary exposure of the Qatari population to food mycotoxins and reflections on the regulation limits

**DOI:** 10.1016/j.toxrep.2019.09.009

**Published:** 2019-09-24

**Authors:** Muna Al Jabir, Andrei Barcaru, Aishah Latiff, Morana Jaganjac, Gouda Ramadan, Peter Horvatovich

**Affiliations:** a Central Food Laboratories, Public Health Department, Ministry of Public Health, Doha, Qatar; b Faculty of Medical Sciences, Laboratory Medicine, Hanzeplein 1, 9713 GZ, Groningen, the Netherlands; c University of Groningen, Department of Analytical Biochemistry, Groningen Research Institute of Pharmacy, Antonius Deusinglaan 1, 9713 AV, Groningen, the Netherlands; d Anti Doping Lab Qatar, Sport City Road, P.O Box: 27775, Doha, Qatar

**Keywords:** Mycotoxin, Risk assessment, Aflatoxin, Ochratoxin, Fumonisin, Margin of exposure, Tolerable daily intake

## Abstract

The article estimates the exposure of the population of Qatar to aflatoxins, fumonisins and ochratoxins, measured in food samples collected from the markets of Doha. The mycotoxin extractions were performed using the “Quick Easy Cheap Effective Rugged Safe” Method (QuEChERS) and the extract measured with LC–MS/MS. High contamination with aflatoxins was detected in Nuts and Spices (e.g. 534.15 ng/g and 371.6 ng/g respectively). The estimated daily intake (EDI) level was estimated using statistical data on average Qatari population. Additionally, the probability to exceed TDI was calculated for fumonisins and ochratoxins. The results indicate high exposure to aflatoxins (with alarming values of margin of exposure, i.e. MoE).

The article points to the necessity of a regular assessment and reevaluation of the concentration limits allowed in food products accounting for statistics on the population (i.e. food consumption), economic growth of the country, and monitoring of mycotoxin contamination of food as much as technological, financial and human resources of a country allows.

## Highlights

• Quantification of mycotoxins using QUEChERS showed elevated concentrations of mycotoxins in food products.
• Estimation of daily intake EDI of the mycotoxins exceeds TDI thus indicating high exposure of the population.
• Probabilistic estimate shows the countries without regulations on mycotoxins are the main source of contamination.
• There is a constant need for updating of regulatory levels of mycotoxins given the updated statistics on population.

## 1 Introduction

Exposure to mycotoxins is known to cause serious illnesses among which are liver cancer, endocrine diseases, chronic gastritis and Reye’s syndrome [1]. Aflatoxins, and in particular aflatoxin B1 (AFB1), are classified as Group I carcinogens by the International Agency for Research on Cancer (IARC) and are linked with the occurrence of the liver cancer and cirrhosis [[2], [3], [4]]. fumonisins are known to inhibit the ceramide synthase and consequently can cause oxidative stress, apoptosis and disruption of the cellular cycle [5]. Exposure to ochratoxin A (OTA) is associated with the development of renal cancer, mitochondrial damage and embryotoxicity [6,7]. Due to the potent carcinogenic and adverse biochemical effects of mycotoxins [3], exposure through food needs to be kept as low as possible. Therefore strict regulations and guidelines have been set by different organizations, such are World Health Organization (WHO) and the European Food Safety Authority (EFSA), in order to ensure consumer safety. The concentration limit for the total aflatoxin (including all quantities of B1, B2, G1, G2 and M1 forms) content is 4 ng/g as described by EFSA. The increase of the allowed limits to 8 and 10 ng/g was discussed by the Scientific Panel on Contaminants in the Food chain (CONTAM) in 2007 would, which concluded that increasing the maximal limit would increase considerably the concentration in consumed food and consequently increase the health risk of the population [8]. For products like peanuts, peanut butter, pistachios, almond - sesame-cotton seeds, sunflower seeds, corn wheat - rice-soybean, GSO standardization (GSO 841/1997) allows 20 ng/g of aflatoxins [9]. For fumonisins (B1 + B2) the maximum allowed level in foodstuff (i.e. maize and maize based products) varies from 800 to 2000 depending on the type of maize products [10].

For OTA, the European Commission set maximum contamination levels to 10.0 ng/g for dried nuts, soluble coffee and spices, 5 ng/g for cereal grains and roasted coffee and 3 ng/g for cereal products [10,11].

Typically used values for the risk assessment are tolerable daily and weekly intake (TDI and TWI). TWI is only used for the risk assessment of exposure to ochratoxin and fumonisin. These values vary slightly from one country to another depending on the type of mycotoxin and on the regulatory organization establishing the tolerable value. It is important to mention here that the toxicity was not evaluated for all mycotoxins and hence, for the most common mycotoxins (i.e. aflatoxins, ochratoxins, fumonisins and zearalenone), the TDI and TWI are often expressed in so called temporary (t-TDI), provisional (PTWI) and maximum provisional (PMTWI) levels [12,13].

This study focuses on the risk assessment and dietary exposure to aflatoxins (with particular emphasis on aflatoxin B1), fumonisins and fchratoxins in Qatar. The TDI levels (fumonisins 2000 ng/kg bw, ochratoxin 14 ng/kg bw) established by the FAO/WHO Joint Expert Committee on Food Additives (JECFA) for the fumonisins and ochratoxins are used [14,15] while for the aflatoxins, who are a class 1 carcinogens, there is no TDI set. The common approach of estimating risk exposure for such carcinogenic compounds is the calculation of the Margin of Exposure (MoE). MoE is calculated using what is known as no-observed-effect level (NOEL), sometimes referred to as the no-observed-adverse-effect level (NOAEL) or, alternatively, the benchmark dose lower confidence limit (BMDL_10_ or BMDL_05_) – which uses the entire dose–response curve from animal studies [16]. Although there is no clear safe value for MoE, a common practice is to use 10.000 or greater which would indicate “low concern” [17].

For fumonisins, the level of TDI established by the European Union Scientific Committee for Food (SCF) and JECFA is 2 μg/kg of body weight (bw) [18].

Ochratoxins have a TDI level of 14 ng/kg bw which brings it closer to the fact that is group 2B carcinogen.

Leblanc et al. estimated the dietary exposure to principal mycotoxins for the first French Dietary Study [12]. A vast number of publications are available describing risk assessment strategies for mycotoxins in foods [[19], [20], [21], [22]] across the world. Tsakiris et al. assessed the risk of children’s exposure to aflatoxin M1 residues in milk collected from the markets of Grece [23]. Solfrizzo et al. evaluated the probable daily intake (PDI) from the samples of urine collected from the population of southern Italy [24]. There are several previous surveys on the occurrence of these three types of mycotoxins in food products available on the markets in Qatar [[25], [26], [27], [28]]. In 2004, Abdulkadar et al, listed alarming numbers of highly contaminated products with the major mycotoxins [26]. According to Abdulkadar et al, 28 (out of 106 total) samples had measured concentrations of aflatoxins between 140–81640 ng/kg with the highest levels in pistachios followed by the chili powder. The study also found 11 samples contaminated with ochratoxin having the concentration in the range of 200–4910 ng/kg. The products that yielded highest levels of ochratoxin were chili powder, rice and grape raisins. A recent survey of mycotoxin occurrence in baby food from the Qatari markets [28] showed significant number of samples with the concentration above the acceptable levels set by EU. More specifically, for the noodles, the most contaminated food category, 33% of the samples were identified to be contaminated with OTA having levels above 500 ng/kg, 25% of the samples had levels higher than EU level for AFB1 (i.e. higher than 100 ng/kg for processed cereal-based foods and baby foods for infants and young children). Although thorough and informative, these studies do not include the actual exposure risk of the population, i.e. estimation of the daily intake of particular food category with respect to TDI and PTWI and evaluation of MoE. An important aspect of risk assessment study is the dietary habit of the population. Specifically the difference between the consumption patterns of the region of assessment (i.e. Qatar) and the region where the regulatory limits originated (i.e. Europe). To the authors’ knowledge there are no previously reported studies that would consider the change in the quality of life of the population of Qatar to re-evaluate the maximum allowed mycotoxin levels.

In the present work we were aiming (i) to assess an up-to-date mycotoxin (i.e. aflatoxin, fumonisin and ochratoxn) contamination levels of various food products acquired in markets of Doha (Qatar); (ii) to assess the exposure to these three types of mycotoxins in Qatari population and (iii) to assess the validity of the concentration limits, for the population of Qatar, taking into consideration the latest state of the dietary habits of the Qatari population. In this study, the levels of 3 mycotoxins in forms provided by producing fungi (AFB1, AFB2, AFG1, AFG2, FB1, FB2, OTA and OTB) foodstuff were assessed using QuEChERS-based LC–MS/MS method [29], and only intact form of mycotoxins were used.

## 2 Materials and methods

The method used for this work is thoroughly discussed in a previous publication from Ramadan et al. [30]. An overview of the same method is briefly described below.

### 2.1 Reagents and chemicals

LC–MS grade methanol, formic acid and QuEChERS [41] kit were purchased from Sigma Aldrich. Deionised water, water purified through Milli-Q system (Millipore) was used to prepare the standard solutions and for LC/MS analysis. The following mycotoxin standards were used: aflatoxins mix of AFG1, AFB1, AFG2, AFB2 (10 mg / L), ochratoxin A (OTA, 10 mg / L), ochratoxin B (OTB, 10 mg / L) fumonisin B1 (FB1, 50 mg / L), fumonisin B2 (FB2, 50 mg / L) were procured from Sigma Aldrich.

### 2.2 Sample collection

The samples of spices, nuts, cereals, grains and dried nuts were collected from Qatari local markets in a clean, dry, leak-proof containers, securely sealed and their origin recorded (Supplementary material A). A total of 401 samples were collected in the period from December 2016 – July 2017. The samples were stored in opaque containers to reduce exposure to the light which can affect the analytical results. Incremental samples were collected together into one container to make up the samples to be sent to the analyst. When sampling from retail outlets, it was made sure that enough packs have been taken to give an aggregate sample representative of a batch picked randomly from the same batch. The collected samples were dispatched to laboratory as soon as possible, ensuring they are in good condition. The pre packed retail samples were not removed from their packaging. Entire pack was sealed in a plastic bag after purchase. All samples were stored in a cool & dark place. Table A in the supporting information (Supporting Information A) describes the number of samples collected from the different food category with additional details. Samples and food samples were stored at -20 °C and 4 ± 2 °C respectively until analysis.

### 2.3 Extraction procedure

Homogeneous food samples (5 ± 0.05 *g*) were weighed in 50 mL PTFE tubes (extraction kits). A mixture of 20 ml of methanol-water solution (80/20 v/v) was added and mixed vigorously for 1 min. QuEChERS buffer solution containing 1 *g* sodium citrate, 0.5 *g* sodium hydrogen citrate sesquihydrate, 4 *g* magnesium sulfate and 1 *g* sodium chloride was added to the sample mixture. The mixture in the PTFE tube was shaken vigorously for 1 min and centrifuged at 10,000 RPM for 10 min. Eight ml of upper clear solution was transferred into dispersive solid phase extraction tube (15 mL Polyethylene tube) containing 150 mg primary secondary amine (PSA) and 900 mg anhydrous magnesium sulphate. The tube was capped and the extract vigorously mixed with the sorbent for 1 min and then centrifuged at 4000 RPM for 5 min. Two mL of the clear extract was transferred into HPLC vial and 20 μL injected for LC–MS/MS analysis.

### 2.4 LC–MS/MS analysis

High performance liquid chromatograph (Agilent 1290 infinity series) coupled to mass spectrometer (Agilent LC–MS/MS 6470) equipped with electrospray ion Source (ESI) and operated with MassHunter data acquisition software was used for targeted multiple reaction monitoring analysis of mycotoxins. ZORBAX Eclipse XDB-C8 column was used with 4.6 × 150 mm of ID and length and particle size of 5 μm. All extracts were chromatographically separated maintaining the column temperature at 23 °C and using gradient elution at 0.6 mL/min, in which mobile phase A was composed of deionized water with 5 mM ammonium formate and 0.2% formic acid and mobile phase B consisted of methanol with 5 mM ammonium formate and 0.2% formic acid. MS was operated in the positive ESI mode and the data acquisition was based on multiple reaction monitoring (MRM) of the analytes using parameters shown in the Table 1.

**Table 1:** Multiple Reaction Monitoring Transitions for Mycotoxins.

ANALYTE TRANSITIONS							
No.	Compounds	RT	Precursor Ion(m/z)	Product Ion(m/z)	Fragmentor voltage	Collision energy	Ionization mode
1	AFG2	3.03	331.2	313.1/245.1	160	23/30	Positive
2	AFG1	3.96	329.2	311.1/243.1	150	20/25	Positive
3	AFB2	5.07	315.2	287.1/259.1	160	24/30	Positive
4	AFB1	6.45	313.2	285.1/241.1	160	22/38	Positive
5	OTB	13.2	370.0	205.0/324.0	100	10/19	Positive
6	FB1	15.2	722.4	352.2/334.0	200	35/40	Positive
7	OTA	16.8	404.0	358.0/239.0	90	10/25	Positive
8	FB2	17.3	706.4	336.2/318.3	200	35/35	Positive

One transition ion product was used for quantification (Quan) and the other for confirmation (Qual). Peak detection and quantification was performed using MassHunter software from Agilent and using 3 S/N as peak picking parameter. The identity of Mycotoxin compounds in an extract was considered to be confirmed if (1) the retention time of the compound peak matched the peaks of the Mycotoxin standard in neat solution with tolerance of ±0.15 min, and (2) the ion ratios of the quantifier and qualifier product ion of the measured compounds was within ±20% of the corresponding product ion ratio of the Mycotoxin standard. Once the presence of a Mycotoxin compound was confirmed in an extract, the concentration of the residue was obtained from the appropriate calibration function which is the matrix-matched calibration standards (Supporting Information C) using Quan transition. Calibration standard curves were generated by plotting the peak areas for each Mycotoxin compound with Quan transition versus its concentration in the matrix-matched standard solution and used for the quantification of each compound in the sample extract. The matrix-matched calibration curves included six concentration levels for all target analytes (Supporting information C). In case of low concentrations, the top 3 calibration points were excluded from the calibration curves, whereas in the case of high concentration levels, the 3 lowest calibration points were excluded from calibration curves. A minimum of 3 calibration points were used for quantification. The standard curves for each Mycotoxin compound were linear with correlation coefficients greater than 0.995. Samples that had higher concentration than the highest level in the calibration curve were diluted to fit in the middle range of the calibration range using with deionized water with 5 mM ammonium formate and 0.2% formic acid (eluent A used for LC–MS/MS analysis).

### 2.5 Risk assessment

To estimate the level of mycotoxins for the groups of food consumed by an average person in Qatar, the following equation was used:

(1) $$EDI=\frac{Amount\times \rho \;\times \bar{Rt}}{Body\;Weight}$$

where, “Amount” defines the quantity of the consumed food group in g per day by one person, $\bar{Rt}$ denotes the average of the detected mycotoxins concentration in ppb in the food products ($\mu g\times {kg}^{-1}$), and EDI is the estimated daily intake of a mycotoxin for one food group per body weight per day. ρ is the estimated ratio for the analyzed food subgroup (i.e. fraction of surveyed food subgroups in a specific food category). To exemplify, if the survey of spices contains 3 sub-groups of spices: “salt, curry, red paprika” and the consumed amount evaluated by the survey is *X*. For a mycotoxin study detecting mycotoxin only “curry” and “paprika” sub groups then ρ=2/3  of the surveyed spices. This, of course is only an estimate and may deviate from the actual consumption of the relevant sub-categories.

*For non-carcinogenic compounds* like fumonisins and ochratoxins, EDI must be compared to the TDI values set by JECFA to quantify the risk of dietary exposure. Tresou et al. [31] included a value of probability to exceed a fixed safe reference limit d (i.e. TDI in this case):

(2) $$p\left(K_{i}\geq d\right)=\frac{\#\left(K_{i}\geq d\right)}{N}$$

In Eq. 2, $K_{i}$ signifies the calculated individual exposure, with i=1,2,…,N measurements of the same food group from N different sources, while d signifies the hazardous quotient (i.e. TDI or TWI depending on the case study). From the Eq. 1 it is easy to estimate that the higher the amount of the consumed product and the higher the concentration of the mycotoxins, the higher is the exposure EDI. The values for d are listed in the introduction. By a simple derivation of EDI with respect to the “body weight” (bw) it is straight forward that the small fluctuations in the weight of the adult consumer’s body, plays a minor role in the exposure estimation of the population. Hence, a convenient approach is to work with an average value of the population body weight. In the current work detailed information on the body weight (i.e. devised by gender and age) of Qatari males and females is used for the calculation of the EDI and an average body weight is used for computing $r\left(d\right)$.

*For carcinogenic compounds* like AFB1, the Margin of Exposure (MoE) was used to assess the risk of exposure for aflatoxins. MoE is calculated as follows:

(3) $$MoE=\;\frac{BMDL_{\%}}{EDI}$$

Here, ${BMDL}_{\%}$ ($\frac{\mu g}{kg_{bw}day}$) is the bench-mark dose lower confidence limit with the index % varying between 1,5 and 10 percent of the response curve obtained from animal studies. Higher EDI results in lower MoE thus the higher is the risk. A value of 10,000 or higher would indicate a “low concern”. MoE is at the time, the most used mean of assessment of carcinogenic compounds. However, the limits of MoE are strictly linked to animal studies which can often be misleading as the animals may be more resistant to aflatoxins than humans.

In 1974 Wogan et al. [32] published values for $BMDL_{10}=0.251$, $BMDL_{05}=0.156$ and $BMDL_{01}=0.053$ (in $\frac{\mu g}{kg_{bw}day}$) which were proven to be reliable by Benford et al. [16]

Having the information on average food consumption and an average person body weight, one can estimate the quantity of the intake of mycotoxins for an average house hold as it is expressed in Eq. 1. Al-Thani et al. published an overview of food patterns and diet quality in Qatar [33] which can be used to estimate the average quantity of the type of foods consumed by the average Qatari population. WHO published in 2013 a report regarding chronic disease risk factor surveillance [34], where the data on average body weight of Qatar population is available.

All the calculations for the risk assessment were carried out in Python 3 using Anaconda distribution in PC equipped with Intel Corei7 7700k processor and 64 GB of RAM. An example of calculation for aflatoxin B1 is included in a form of an Excel file as supporting information A. [35]

## 3 Results and discussion

### 3.1 Food analysis results

Method validation was carried out on pistachio, rice and chili powder samples as representative matrices for nuts, cereals and spices respectively (Supporting information B). The limit of detection is estimated as three times the standard deviation of sample blanks fortified at the lowest acceptable concentration level. The limit of detection (LOD) were found to be in the range of 0.01-0.06 ng/g for aflatoxins, 0.025-0.051 ng/g for ochratoxins and 0.52–1.00 ng/g for fumonisins depending on the food type. The quantification method was found to be linear (with correlation coefficient ≥ 0.99) for all the mycotoxins, from the LOQ 0.5 ng/g up to 50 ng/g for aflatoxin and ochratoxin compounds and from 10 ng/g up to 1400 ng/g for fumonisins.

Repeatability of mycotoxin quantification were carried out using 7 replicates of spiked samples at different concentration levels in grinded pistachio, rice and chili powder samples respectively. The CV% (RSD%) for the three different levels was lower than 20%. Reproducibility was estimated from validation data by pooling the variances of the different levels; the pooled CV% for reproducibility was lower than 10%. The recovery for all mycotoxins was within 80–120%.

The results of the food analysis are summarized in Table 2. According to the JECFA and WHO protocol, the values marked “<LOQ” (i.e. below LOQ) are replaced with the half of the corresponding LOQ value [15]. The values marked with “ND” (i.e. Not Detected) are replaced with 0. This is a common practice mentioned in other relevant references [12,13,31,36]. In Table 2, N indicates the number of analyzed samples, % rejected is the fraction of the samples that were labeled “Rejected” due to elevated concentration of a certain mycotoxin.

**Table 2:** Overview of the samples analyzed and the concentrations of major mycotoxins in ppb (* values were considering “< LOQ” as half the LOQ value).

Sample group	N	Samples above the limit (%)	Contaminated samples			
			Mycotoxin	Range (ng/g)	Positive (%)	Geometric Mean (ng/g)
Cereals	52	5.70	aflatoxin total	0.25 - 0.6	6	0.237
			FB1	5 - 1731	29	19.86*
			FB2	5 - 675.4	21	10.41*
			OTA	11.04	2	11.04
			OTB	0	0	0
Dried Fruits	54	0.00	aflatoxin total	8.53	2	8.53
			FB1	5.00 - 481.4	9	16.44*
			FB2	5 - 74.31	9	8.57*
			OTA	10.11 – 140	4	37.62
			OTB	0	0	0
Grains	35	0.00	aflatoxin total	0	0	0
			FB1	5	9	5*
			FB2	5	9	5*
			OTA	0	0	0
			OTB	0	0	0
Nuts	119	2.50	aflatoxin total	0.29 - 534.2	7	5.67
			FB1	5 - 17.11	8	5.73*
			FB2	5	7	5*
			OTA	4.25 - 17.33	3	12.19
			OTB	0	0	0
Spices	141	11.3	aflatoxin total	0.25 - 371.6	64	5.38
			FB1	5 - 955.8	17	22.05*
			FB2	5 - 141.3	15	10.48*
			OTA	5.02 - 10.69	8	8.6
			OTB	0	0	0

As in the study of Abdulkadar, the results of the current study point to the fact that the “Nuts” and “Spices” have the highest content of aflatoxin (i.e. maximum values are 532.2 and 371.6 ng/g respectively). The values however exceed the report of Abdulkadar et al and Hammami, Fiori et al in both food categories. The aflatoxin level in “dried fruits” were the closest to the level reported in the previous studies.

For fumonisin B1, the highest levels were detected in “Cereals” in particularly in “Corn Whole” with 1731 ng/g followed by the same sub-category with 1439 ng/g (see supporting information). For Nuts (sub-category “Almonds peeled”) the level of fumonisin B1 is 17.11 ng/g. In case of ochratoxins, only OTA was detected predominantly in “*nuts”* and “*dried fruits”* (approximately 3.7% of the samples were positively labeled for OTA).

### 3.2 Risk assessment

The food groups and the corresponding consumption amount evaluated by Al-Thani et al are indicated in Table 3. The groups irrelevant for the current mycotoxin study were excluded (such as Meat, poultry & fish; Milk, dairy products & eggs; Tubers, Oils & Fats; Sugar & sweets, Beverages etc.).

**Table 3:** Food pattern described in [33]. The values represent the food intake level in g/day. The underlined subcategories are listed in the analyzed sample categories.

Food Group	Included in group	Qatari	Non-Qatari	Total	Estimated ratio for the analyzed food subgroup (ρ)
		Amount (g)	Amount (g)	Amount (g)	
Cereals and cereal products	Different types of rice, wheat and wheat products, different types of bread, macaroni, cakes, biscuits and oriental sweets	442	279	366	1
Nuts and seeds	Peeled and unpeeled types of nuts, salt, spices, pickles, chicken and beef stocks	28	22	27	1/3
Fruits and vegetables	Fresh, dried and canned fruit	599	463	571	1/3

It is worth mentioning that the “*fruits and vegetables*” category in Table 3 includes 3 sub-categories, namely “*fresh”, “dried”* and *“canned fruits”* out of which, *t*he analytical study covers only “*dried fruits”*. Thus a best estimate of the consumption of only *dried fruits* is a fraction (i.e. 1/3) of the surveyed consumption of “*fruits and vegetables*”. The survey of the consumption of “*nuts and seeds* “covers 6 sub-categories out of which only 2 were included in the analytical study. In this case, the best estimate of food consumption is 2/6 of the amounts displayed in columns 3–5 with headers Qatari, Non-Qatari and Total and corresponding to the category *“nuts and seeds”*. All estimated ratios (ρ) are included in Table 3. For the *EDI* estimation, only the column 5 Total is used as it covers the consumption of the overall population.

The average bodyweight data is available in Table 4.

**Table 4:** Bodyweight estimation for Qatari population according to age range and gender [34].

Age group (years)	Men		Women	
	Weight (kg)	95% CI	Weight (kg)	95% CI
18-44	84.7	82.6-86.7	71.2	69.6-72.8
45-64	84.2	81.9-86.5	80.5	78.3-82.7
18-64	84.6	82.8-86.3	73.4	72.0-74.9

The analyzed samples were labeled with “Accepted” or “Rejected” (food allowed or not allowed to be marketed following regulatory control) depending on the concentration of the analyzed mycotoxins. If the level of at least one type of mycotoxin exceeds the levels established by GSO (in case of aflatoxin) or EC (for the rest of the mycotoxins), the food product is labeled as “Rejected”. These level however may not reflect the fitness for human consumption and for this reason, in this study, we calculated the EDIs for all analyzed samples and subsequently only for the samples labeled “Accepted”. This gives a better view on the efficiency of the applied regulations. The discussion is further split in “Risk assessment for Aflatoxin B1″ (due to its potent carcinogenicity) and “Risk assessment of fumonisins and ochratoxins”. For the former two mycotoxin, as it is indicated in chapter 7 of the 2012 IARC monograph [18], there are no convincing evidences to link these compounds with human disease. For this reason, only TDI values set by JECFA to assess the risk for human exposure to fumonisins (B1 + B2) and ochratoxin (A + B) were used.

#### 3.2.1 Risk assessment for aflatoxins B1

The detailed estimations provided in the supporting information (SI) table of Appendix A indicate that the highest risk comes from the “*Nuts and Spices*” for which there are values of aflatoxins B1, B2 and G1 concentrations that considerably exceeded the allowed levels by GSO (i.e. 20 ng/g). Estimated daily intake combined the concentration levels found in several food categories, and the data from Al-Thani et al [33] and [37].

For AFB1, *EDI*s are shown in Table 5. Considering the BMDL values from Wogan et al. from 1974, the MoE values shown in Table 5 do not meet the safety level (i.e. > 10,000). Even after removing the samples that are above the allowed concentration set by GSO, the MoE did not rise considerably.

**Table 5:** EDI and MoE values for AFB1. The values of MoE and EDI are calculated alternatively only for the samples labeled with “Accepted” to indicate the insignificant improvement in the margin of exposure.

Gender:	Men			Women		
Age (years)	18-44	45-64	18-64	18-44	45-64	18-64
EDI of **AFB1** (ng/kgbw/day)	17.39	17.5	17.41	20.7	18.3	20.07
MoE (BMDL_01_)	3.04	3.02	3.04	2.56	2.89	2.64
MoE (BMDL_05_)	8.96	8.91	8.95	7.53	8.52	7.77
MoE (BMDL_10_)	14.42	14.34	14.41	12.12	13.71	12.5
EDI of **AFB1 labeled “Accepted”** (ng/kgbw/day)	16.63	16.73	16.65	19.78	17.49	19.19
MoE (BMDL_01_)	3.18	3.16	3.18	2.67	3.02	2.76
MoE (BMDL_05_)	9.38	9.32	9.36	7.88	8.91	8.12
MoE (BMDL_10_)	15.09	15.00	15.07	12.68	14.34	13.07

It is possible to estimate potentially maximum allowed concentration of AFB1 in Nuts and Spices, having fixed the BMDL01 value, knowing the consumption amount and body weight of an average person. The max concentration estimate is:

(4) $$\bar{R}<\frac{\text{min}\left(Bw\right)\times 10^{-4}\times BMDL_{01}}{\text{max}\left(Amount\right)}$$

**Fig. 1:**
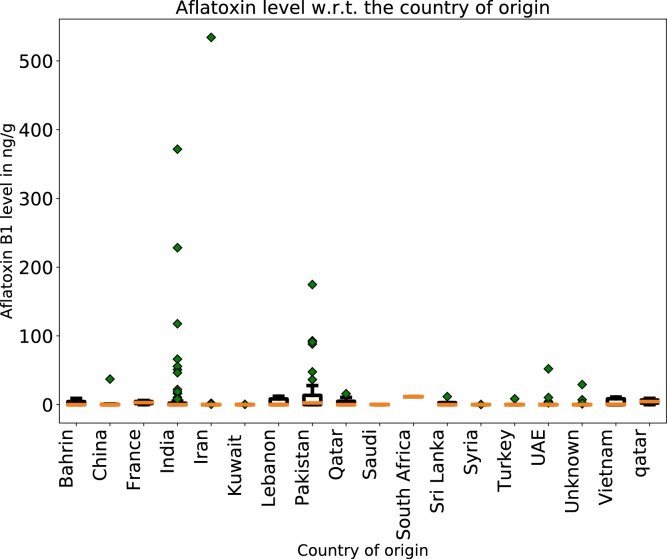
Boxplots for the concentration of aflatoxin B1 with respect to the countries of origin. The yellow line indicates the average of the distribution of the concentrations. The green markers indicate outliers from the distribution. In this case however the outliers are the most contaminated samples and are of high interest.

Here, the amount is taken at its maximum only when a range of consumption amount is available. Further, the consumption and body weight values shown in Table 3, Table 4, and BMDL01 of 0.053 μg/kg bw /day are introduced in Eq. 4. The value for the bw is taken from the lowest limit of the confidence interval of the minimum body weight value displayed in Table 4 (i.e. the lower bound bw value corresponding to the average female from the age range of 18–44 years is 69.6 kg). Thus one can deduce the maximum value of $\;\bar{R}\;<\;0.04\;ng/g$ for nuts and spices, $\bar{R}\;<\;0.002\;ng/g$ for cereals and $\bar{R}\;<\;0.002\;ng/g$ for “*dried fruits”*. All calculated amounts are significantly lower than the LOQ/2. On one hand, this values indicate that the analytical methods should be much more sensitive for a more objective evaluation of the risk (a much lower LOQ would be required) and on the other hand the values of 20 ng/g (the maximum level allowed by GSO) is extremely high for a compound like AFB1. Even the values set by European Commission for AFB1, ranging from 0.1 ng/g (for baby food for infants and young children) to 0.6 ng/g (for dried figs) are extremely high compared to estimated maximum $\bar{R}$ using Eq. 4. According to United Nations COMTRADE database on international trade, Qatar increased, from 1998 to 2017, the importations of *“coffee, tea, mate and spices”* approximately 8 folds. Similar increase in importation can be seen for *“Cereals”* as well. These products are known to be most often contaminated with mycotoxins, and aflatoxins in particular. The highest part of the importation of these products derives from United Arab Emirates (UAE), i.e. 36%, followed by India with 21%, for *“coffee, tea, mate and spices”.* For *“Cereals”*, the highest import part originates from India with 38% followed by Russia with 13%. [38] Fig. 1 indicates the countries of origin of the products that yielded at least one sample contaminated with AFB1. The products found in the markets of Doha, produced by Pakistan, India, China and UAE are the most contaminated with AFB1.

In 2003, a FAO report [39] was published indicating that Pakistan, at the time of the publication of the report, did not have regulation limits for mycotoxins in foods, dairy products and feed. India however does have regulatory limits, as indicated in the same report from FAO and in a review published by Anukul et al. in 2013 [40]. The allowed maximum concentration of AFB1 however, for India, is 30 ng/g which is extremely high given the daily consumption of an average Qatari person and the carcinogenicity of this toxin.

#### 3.2.2 Risk assessment for fumonisins and ochratoxins

For the fumonisins (Table 6), the estimated exposure is below the TDI value set by JECFA. The evaluation of the samples labeled “Accepted” show a considerable reduction in the ratio between EDI and TDI, i.e. from 13% to 3.3% for males age 18 to 44, and from 15% down to 4% for females age 18 to 44. The latest indicate high efficiency of the regulatory control of the concentration in case of fumonisins. For this toxin, as in the case of AFB1 and AFG2, the highest risk exposure is due to the consumption of “*Spices*” and “*Nuts*” (see supporting information, appendix A).

**Table 6:** Total estimated intake level of fumonisins. EDI /TDI ratio is calculated using a TDI of 2 μg/kg bw.

Gender:	Men			Women		
Age (years):	18-44	45-64	18-64	18-44	45-64	18-64
EDI of **fumonisins** (ng/kgbw/day)	261.65	263.21	261.96	311.27	275.31	301.94
EDI/TDI:	0.13	0.131	0.13	0.155	0.13	0.15
EDI of **fumonisins Labeled “Accepted”** (ng/kgbw/day)	67.67	68.07	67.75	80.5	71.2	78.08
EDI/TDI:	0.033	0.034	0.033	0.04	0.035	0.039

The EDI for ochratoxins (Table 7), exceeded the TDI by a factor of 1.65 for the minimal EDI (i.e. males under 45 years) and by a factor of 1.97 for the maximum EDI (i.e. females, age under 45 years).

**Table 7:** Total estimated daily intake level of ochratoxins. EDI/TDI ratios are calculated using a TDI of 14 ng/kgbw/day.

Gender:	Men			Women		
Age:	18-44	45-64	18-64	18-44	45-64	18-64
EDI of **ochratoxins** (ng/kgbw/day)	23.19	23.32	23.21	27.58	24.4	26.76
EDI/TDI:	1.65	1.66	1.65	1.97	1.74	1.91
EDI of **ochratoxins labeled “Accepted”** (ng/kgbw/day)	22.76	22.9	22.8	27.08	23.95	26.27
EDI/TDI:	1.62	1.63	1.62	1.93	1.71	1.87

The set TDI values for ochratoxins set by JECFA refer to the summation of both OTA and OTB. OTB was not detected in the studied samples, thus ochratoxin level is due to OTA. The high levels for *EDI* result from high consumption of “*dried fruits*” – a type of food that is often contaminated with ochratoxins. Evaluation of the risk for the samples labeled “Accepted” (Table 8, bottom rows) showed that the levels of *EDI* significantly exceeded the TDI level shown in Table 1. This could be the result of exceedingly high allowed levels for the ochratoxins in foods given the consumption of the products in which this toxin is often found. Another reason is a high LOQ, of the analytical approach which increases the uncertainty of the analysis.

**Fig. 2:**
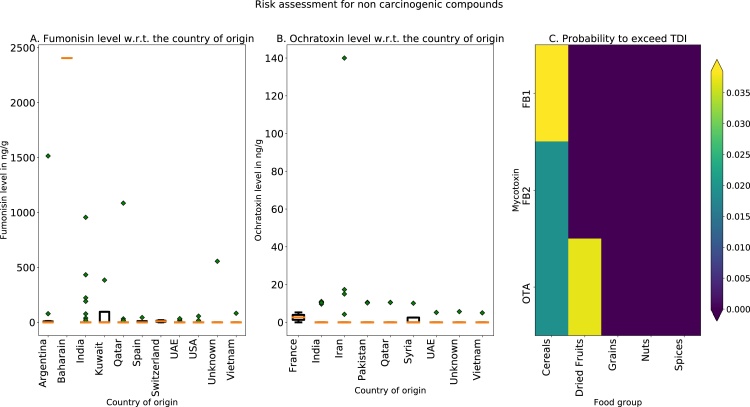
Risk assessment for fumonisins and ochratoxins. The boxplots of concentrations with respect to the country of origin for fumonisins B1 + B2 (A) and ochratoxins OTA + OTB (B). The probability (Eq. 2.) to exceed TDI (C) for fumonisins and ochratoxins using specific food category.

Eq 2 was used to calculate the probability if fumonisins and ochratoxins exceed TDI (i.e. (EDI≥TDI)). Fig. 2 C illustrates the p(EDI≥TDI) for each food group of the current study. In this calculation, the lowest limit of the 95% confidence-interval (CI) of body weight was used as to ensure the most sensitive case or worst-case scenario, i.e. 69.6 kg for females under 45 years. For fumonisins (B1 and B2) and OTA, consumption of cereals yield highest probability to exceed TDI. For OTA, probability to exceed TDI comes also from *“dried fruits”.* The magnitude of the probability is relatively low, however the ratios between EDI and TDI are significantly high. These aspects indicate that there are only a small number of samples contaminated with high concentration of OTA.

Concerning the country of origin of the products contaminated with OTA, highly contaminated products can come even from the countries that have strict regulations (i.e. France, Fig. 2.B). The evaluation of the relationship between the country of origin of particular products and the level of mycotoxins found in those products does not necessarily suggests that the contaminated product is imported. The contamination can occur at any stage of the storage of the product (i.e. storage in the country of origin, transportation, storage in Qatar, etc). However, it is of outmost interest to consider a high probability of contamination in the context of high imported fraction of food available in Qatar. More specifically, high availability of the contaminated products, such as grains and spices, and large consumption amounts of these products suggests high probability to exceed TDI and consequently large risk for the population health. To this end, a constant update of the regulatory limits must be carried out. The latest information on population consumption patterns, overall statistics on the population BW, and economical status of the country are most important in the re-evaluation of the regulatory limits.

Following a similar strategy described in case of AFB1, we can estimate the maximum allowed concentration given the recent data on the consumption and bw. Concretely, the following equation can be used:

(5) $$\bar{R}<\frac{\text{min}\left(Bw\right)\times TDI}{\text{max}\left(Amount\right)}$$

Thus the estimated maximum average concentration to be allowed, for “Cereals” is $\bar{R}<2.66\;ng/g$, for “nuts and spices” $\bar{R}<108.2\;ng/g$ and for “dried fruits” is $\bar{R}<5.12\;ng/g$. For the cereal products, the estimated maximum concentration is close to the level set by the EU for the same food category (i.e. 3 ng/g). For “spices”, the EU commission set values above 15 ng/g which is below the estimated value using Eq. 5 and as such does not require a modification. For “dried fruits”, EU commission sets 5 ng/g limit only for a subcategory, i.e. “dried vine fruits” which is close to the estimated value using Eq. 5 – i.e. 5.12 ng/g.

## 4 Conclusion

The risk assessment analysis using the measured mycotoxin concentration in the food commercialized in Qatar indicates that there is a high exposure to AFB1 and OTA. The same statistical analysis showed that the exposure to fumonisins are below the TDI. The existing level of concentration defined by the GSO in 1997, is extremely high given the consumed quantities of food by the average Qatari in the recent years. An estimation of the MoE level for AFB1 in food was calculated to be below 1 ng/g by two-three orders of magnitude such as 1–10 pg/g. The products with highest amount of AFB1 were imported from Pakistan and India – countries known for lacking in strict regulations of mycotoxin level in foods or having higher permitted levels for concentration of aflatoxins in foods. The method indicates that a constant update of the regulatory limits is needed in concordance with the latest population statistics (i.e. weight and consumption of the average person, dietary habits of subpopulation such as age categories and social classes), the economic development of the country (i.e. availability of certain products, price of the products and imported fraction of the food products from countries without regulations on mycotoxins) and the mycotoxin concentration in different food subcategories. It is however evident that such a dynamic regulatory system would require a complex management system and constant implication from the governmental institutions. An important step towards solving this issue is a close, multidisciplinary collaboration of relevant institutions (i.e. Planning and statistical authority of Qatar, Ministry of Public Health and several research centers from Qatar that could potentially provide a high quality feedback on the exposure risk).

The probability to exceed the tolerable daily level (Fig. 2 C) points to the “*Cereals*” and “*Dried Fruits*” as a source of exposure risk. A more thorough study is needed to estimate the statistical confidence intervals for the *EDI* values (Eq. 1). The current risk assessment based on *EDI* can be improved by using a more precise population statistics on food consumption based on food consumption questionnaires, using statistics on food processing technology used by Qatari population and data on how these food processing technologies alter mycotoxin content. Additional improvement can be achieved by including additional information from the risk assessment with exposure estimation of population using mycotoxin concentration measured from urine and serum.

## Source of funding

Funding for this work was provided by the Qatar National Research Fund (QNRF) under the National Priorities Research Program, with project NPRP8-1472-3-290.

## Conflict of interest

The authors declare no financial relationship with the organization funding this research and no financial interest in the publication of the present research. The data associated to this research is owned by the members of this research projects and the authors of this article. The authors allow the journal to review their data if requested.

## Declaration of Competing Interest

The authors declare that they have no known competing financial interests or personal relationships that could have appeared to influence the work reported in this paper.

The authors declare the following financial interests/personal relationships which may be considered as potential competing interests:

None.
